# Revision and validation of the prosocialness scale for adults (PSA) among chinese college students

**DOI:** 10.1186/s40359-023-01124-3

**Published:** 2023-04-19

**Authors:** Qisheng Zhan, Su Wang, Chengze Li, Mengri Li, Dan Liu, Wei Peng, Fanglin Song, Tongxiao Shi, Yonghui Li

**Affiliations:** 1 School of Education, Tianjin University, Tianjin, China; 2 Institute of Psychology, Tianjin University, Tianjin, China; 3 Psychological Counseling Center, East China University of Science and Technology, Shanghai, China

**Keywords:** Prosocial behavior, College students, Questionnaire revision, Reliability, Validity

## Abstract

**Background:**

Although prosocial behavior plays an important role in the development of individuals, there are few prosocial measurements for college students. This study examines the applicability of the Prosocialness Scale for Adults to a sample of Chinese college students and provides a measurement tool for prosocial behavior among Chinese college students.

**Methods:**

Three sub-studies were conducted in this study to revise the Prosocialness Scale for Adults (PSA) and verify its applicability in Chinese college students. In Study 1, the translated Prosocialness Scale for Adults (PSA) was used to test (N = 436). In Study 2, confirmatory factor analysis was carried out (N = 576). The Scale of School Adjustment for College Students, the Scale of Regulatory Emotional Self-Efficacy, the Prosocial Tendencies Measure, and the Chinese Big Five Personality Inventory were used to test the concurrent validity. And the internal consistency reliability of the scale was tested. In Study 3, the test-retest reliability of the scale was tested 4 weeks after the completion of Study 2.

**Results:**

The results show that the scale has a good single-factor structure (χ2/df = 4.180, CFI = 0.936, TLI = 0.922, GFI = 0.937, IFI = 0.937, NFI = 0.919, AGFI = 0.907, RMSEA = 0.074, SRMR = 0.042). The total score was positively correlated with the scores of the Scale of Regulatory Emotional Self-Efficacy (r = 0.394, p < 0.001), the Scale of School Adjustment for College Students (r = 0.429, p < 0.001), the Chinese Big Five Personality Inventory (r = 0.456, p < 0.001) ,and the Prosocial Tendencies Measure (r = 0.619, p < 0.001). The internal consistency reliability was robust (α = 0.890) and the test-retest reliability was 0.801.

**Conclusion:**

These studies show that the Chinese version of the Prosocialness Scale for Adults (PSA) has good reliability and validity and can be used to measure the prosocial behavior of Chinese college students.

**Supplementary Information:**

The online version contains supplementary material available at 10.1186/s40359-023-01124-3.

## Background

Prosocial behavior refers to behavior that is expected to benefit others or society, whether for the purposes of compassion, charity, sharing, assistance, donation, disaster relief, and self-sacrifice [1]. The cultivation of college students’ prosocial behavior is an important part of moral education in colleges and universities [2]. However, in the university, there have been a series of vicious events, such as the sensational “Yao Jiaxin case” and “Lin Senhao poisoning case”, which caused a huge social response. With the emergence of these vicious events, as a matter of fact, they have promoted the social thinking about the moral and prosocial behavior of college students.

Cultivating and shaping prosocial behavior can restrain aggressive behavior and play an important role in individual healthy development and social adaptation [3]. Some studies have shown that thematic education and training on prosocial behavior can increase prosocial behavior and reduce antisocial behavior [4]. In addition, prosocial behavior can affect an individual’s self-esteem by achieving self-satisfaction [5, 6]. Prosocial behavior can affect interpersonal relationships, help improve interpersonal communication, and promote interpersonal adaptation and harmony [7]. In universities, college students’ prosocial behavior is related to their school adaptation. Previous studies have shown that there is a positive correlation between school adaptation and prosocial behavior [8]. Prosocial behavior can positively predict school adaptation [9]. Prosocial behavior is an important factor affecting school adaptation [10].

At present, the main tools to measure prosocial behavior are the Walker McConnell Scale, the Child Behavior Scale, the Akhenbach Child Behavior Scale, and the Prosocial Tendencies Measure and so on. However, throughout the previous research on prosocial behavior measurement tools, the research objects are mainly focused on students below middle school, and less attention is paid to college students [11]. Presently, there is a lack of measurement tools for college students. The level of individual cognition and socialization increases with the increase of age. And as children enter puberty, their prosocial behavior is influenced by emerging interpersonal relationships, cognitive and emotional development, and changes in social environments [12]. Children’s prosocial behavior is mostly based on the principle of reciprocity and moral reasoning, while the prosocial behavior of college students has more characteristics, such as social reciprocity, altruism, public welfare labor, and group restriction [13]. In addition, the dimensional division of these tools is not necessarily suitable for Chinese college students, such as the Prosocial Tendencies Measure, whose dimensions pay more attention to the situation of prosocial behavior rather than prosocial behavior itself and its types [14]. There are some obvious differences and inapplicable contents [15]. Therefore, it is necessary to develop and introduce related tools.

The Prosocialness Scale for Adults developed by Italian psychologist Caprara et al. has good reliability and validity [16]. It has been verified by item response theory (IRT) [17] and can accurately measure and evaluate individual prosocial behavior. Martínez and Gazanfer took adults in Spain and Turkey as samples to verify the localization and application of the scale [18–20]. However, no researchers have introduced this scale in China.

In addition, this scale is developed for adults. As a matter of fact, adults and college students are different. Compared with adults who have entered society, college students have different social environments [21], personality characteristics [22], and moral requirements [23]. Under the influence of such factors, college students will have a different understanding of prosocial behavior. College students who live in the white ivory tower have simpler interpersonal relationships and they are easier to build interpersonal trust. Thus, they are more likely to have a higher level of prosocial behavior [24]. College students believe that the world is fair, which will also lead to a higher level of prosocial behavior. After higher education, college students will have a stronger sense of social responsibility. Also, college students have higher moral requirements for themselves. Maybe that is why their attitudes towards prosocial behavior are more positive [25]. Meanwhile, adults who enter society will have more complex considerations and understanding of the implementation of prosocial behavior. On the basis of the differences between college students and adults, a special scale for college students is required.

Therefore, this study takes Chinese college students as a sample to test the applicability of the adult prosocial scale in Chinese college students and provides a measurement tool for the prosocial behavior of Chinese college students so as to provide a reference for moral education and mental health services in colleges and universities.

## Study 1

### Method

#### Participants and procedures

In this study, participants were selected from a university in Tianjin. 450 questionnaires were sent out by random sampling, and 436 valid questionnaires were recovered after deleting the invalid questionnaires with incomplete information, with an effective rate of 96.89%. There were 246 males (56.4%) and 190 females (43.6%). Their age ranges from 17 to 24 years old, with an average age of 19.36 ± 1.28 years. The academic year included 154 first-year college students (freshmen), 127 s-year college students (sophomores), 91 third-year college students (juniors), and 64 fourth-year college students (seniors).

The researcher emailed the original author, Caprara, who authorized us to revise the Prosocialness Scale for Adults. In this study, the scale was translated and back-translated, and the Chinese version of the scale was formed. Because there may be cross-cultural differences between the context of the original scale and the Chinese version, the translated scale is submitted to experts for review, and the item evaluation form is filled out to test the content validity of the scale. Some items of the scale were deleted according to the results of the expert project evaluation form, project analysis, and exploratory factor analysis.

### Measures

#### Prosocialness scale for adults, PSA

The scale, which was compiled by Caprara et al. in 2005 [17], contains 16 items. The scale is a single dimension and is scored by Likert-5 points (range from 1 ="never” to 5 ="always”). Higher scores indicate a higher level of prosocial behavior. The scale was revised after the authorization of the original author. First of all, according to the Chinese cultural background and language expression habits, 10 postgraduates majoring in psychology, and 2 English experts translated and retranslated the original scale many times while keeping the meaning of the items unchanged. Then, 8 psychological experts (3 professors, 3 associate professors and 2 lecturers) check and modify the professional knowledge, item popularity and understandability indicators, and fill in the content validity evaluation form. Finally, the Chinese version of the Prosocialness Scale for Adults was formed, which was the same as the entry and scoring method of the original scale.

## Results

### Item analysis

Discrimination analysis showed that the differences in the scores for each item between the high-score group (the first 27% of the subjects) and the low-score group (the last 27% of the total score) reached a statistically significant level (P < 0.001). The CR value of each topic reached a significant level (CR > 3). The correlation analysis of the item-total score showed that the correlation between the score of each item and the Pearson product difference of the total score of its subscale was statistically significant, and the correlation coefficient was between 0.587 and 0.732. The average score for each item is near 4, and the skewness coefficient and kurtosis coefficient are between − 1 and 1. The specific results are shown in Table 1.

**Table 1:** Differentiation analysis of PSA, correlation analysis of item-total correlation, and general situation of each topic (N = 436)

Item	t	r	M	SD	Skewness	Kurtosis
1	15.412***	0.713**	4.13	0.718	-0.652	0.764
2	14.649***	0.688**	4.02	0.777	-0.596	0.327
3	15.063***	0.708**	4.09	0.735	-0.533	0.114
4	16.180***	0.674**	4.08	0.829	-0.86	0.826
5	15.708***	0.693**	4.12	0.747	-0.459	-0.304
6	14.674***	0.687**	3.70	0.811	0.060	-0.682
7	15.198***	0.695**	3.85	0.843	-0.451	-0.072
8	13.241***	0.604**	3.71	0.917	-0.223	-0.455
9	13.602***	0.627**	4.12	0.745	-0.506	-0.154
10	15.205***	0.654**	3.99	0.844	-0.757	0.555
11	12.346***	0.587**	3.66	0.961	-0.307	-0.440
12	18.615***	0.732**	3.92	0.820	-0.480	0.177
13	15.945***	0.703**	3.80	0.864	-0.487	0.093
14	14.056***	0.654**	3.72	0.905	-0.460	-0.007
15	15.357***	0.658**	3.78	0.896	-0.433	-0.066
16	15.314***	0.625**	3.61	1.004	-0.257	-0.612

Note: *P < 0.05, **P < 0.01, ***P < 0.001。

In this study, the expert evaluation method is used to evaluate the correlation between items and prosocial behavior. The eight-expert (N = 8) evaluation table is shown in Table 2. Four grades (1 = no correlation, 2 = weak correlation, 3 = strong correlation, 4 = very strong correlation) were used to calculate the adjusted kappa (K) value by I-CVI, in which the Kappa (K) value of item 12 and item 16 was lower (K ≤ 0.74), and item 12 with the lowest K value was deleted first.

**Table 2:** Evaluation form of item content validity (N = 8)

Item	Expert rating								N	*I-CVI*	*P*_*c*_	*K* ^*^	grade
	A	B	C	D	E	F	G	H					
1	4	4	4	3	4	4	3	4	8	1	0.0039	1	Excellent
2	4	3	3	3	3	4	4	4	8	1	0.0039	1	Excellent
3	3	4	4	3	3	3	4	4	8	1	0.0039	1	Excellent
4	3	3	4	4	3	4	4	4	8	1	0.0039	1	Excellent
5	4	3	3	3	3	4	4	4	8	1	0.0039	1	Excellent
6	3	4	3	3	3	4	4	3	8	1	0.0039	1	Excellent
7	3	3	2	3	3	4	3	4	7	0.875	0.0313	0.870	Excellent
8	3	4	3	3	3	3	2	4	7	0.875	0.0313	0.870	Excellent
9	3	3	4	4	4	4	4	4	8	1	0.0039	1	Excellent
10	3	4	3	4	4	3	2	4	7	0.875	0.0313	0.870	Excellent
11	3	3	4	3	2	3	2	3	6	0.750	0.1094	0.720	Good
12	2	2	2	3	2	3	2	4	3	0.375	0.2188	0.200	Bad
13	2	4	3	3	3	3	3	4	7	0.875	0.0313	0.870	Excellent
14	4	2	2	3	4	4	4	4	6	0.750	0.1094	0.720	Good
15	2	4	4	3	3	3	3	4	7	0.875	0.0313	0.870	Excellent
16	2	3	2	3	3	3	2	4	5	0.625	0.2188	0.520	General

### Exploratory factor analysis

Make an exploratory factor analysis of the remaining projects. First, the adaptability of the data was tested. The results showed that the value of KMO was 0.929 and the value of χ^2^ in Bartlett’s test was 2871.542 (P < 0.001), which was suitable for exploratory factor analysis. Principal component analysis and Promax oblique rotation were used to set the extraction feature value to be greater than 1, and exploratory factor analysis was carried out. The results show that the characteristic root of the first factor is 6.721 and the characteristic root of the second factor is 1.250. According to the Hambleton standard, the characteristic root of the first factor is 3 times more than that of the second factor, which indicates that the scale is one-dimensional. The results of the exploratory factor analysis show that the factor loads of item 16 and item 8 are greater than 0.4 on both factors, and the absolute value of the difference between the two factors is less than 0.2. Priority was given to deleting item 16 with a higher load on the two factors. After each item was deleted, exploratory factor analysis was carried out again, item 14 and item 8 are deleted in turn, and finally a model of 12 items is obtained. The final results show that the cumulative variance contribution rate of a single factor is 48.305%, and the factor load of each item is between 0.541 and 0.781. The results are detailed in Table 3. On this basis, the items are renumbered to form the Chinese version of the Prosocialness Scale for Adults, which includes 12 items.

**Table 3:** Exploratory factor analysis of the Chinese version of the Prosocialness Scale for Adults

Item		Loading
1	I am happy to help my friends or colleagues in the activities.	0.781
3	I try to help others.	0.773
5	I am very warm-hearted to those who need help.	0.747
2	I would like to share my things with my friends.	0.738
4	I can help those in need in voluntary activities.	0.727
13	I try to get close to and take care of those in need.	0.697
7	I will try my best to help others avoid getting into trouble.	0.692
6	I will help those in need immediately.	0.688
10	I try to comfort those who are sad.	0.660
9	I would like my knowledge and ability to benefit others.	0.643
15	I will spend time with friends who feel lonely.	0.615
11	I am easy to lend money or other things.	0.541

## Study 2

### Method

#### Participants and procedures

By using the convenient sampling method, 600 questionnaires were distributed in three universities in Tianjin. A total of 576 valid questionnaires were collected after deleting the unanswered and invalid questionnaires with incomplete information, and the effective rate of the samples was 96.00%.

Among them, 295 were males (51.2%) and 281 were females (48.8%). The age of the sample ranges from 17 to 23 years old, with an average age of 19.23 ± 1.24 years. The academic year included 185 first-year college students (freshmen), 170 s-year college students (sophomores), 114 third-year college students (juniors), and 107 fourth-year college students (seniors).

In addition to the revised Chinese version of the Prosocialness Scale for Adults, the Scale of School Adjustment for College Students (SSACS), the Scale of Regulatory Emotional Self-Efficacy (SRESE), the Chinese Big Five Personality Inventory brief version of the agreeableness subscale (CBF-PI-B), and the Prosocial Tendencies Measure (PTM) were used in this study. These scales have been described below.

### Measures

***The Scale of School Adjustment for College Students, SSACS.***

The scale, which was compiled by Hou Jing in 2014 [26], contains 53 items, which is divided into seven dimensions: learning adaptation dimension, teacher-student relationship adaptation dimension, collective adaptation dimension, classmate relationship adaptation dimension, autonomy dimension, life adaptation dimension, and school environment adaptation dimension. The scale is scored by Likert-5 points (ranging from 1 = “completely inconsistent” to 5 = “complete fit”).

Higher scores indicate a higher level of the school adapts. In this study, the Cronbach’s α coefficients of the seven subscales were 0.890 (learning adaptation), 0.841 (teacher-student relationship adaptation), 0.878 (collective adaptation), 0.753 (classmate relationship adaptation), 0.733 (autonomy), 0.828 (life adaptation), and 0.715 (school environment adaptation).

***The Scale of Regulatory Emotional Self-Efficacy, SRESE.***

The scale was compiled by Caprara et al., and translated by Chinese scholar Wang Yujie et al. in 2013 [27]. It contains 17 items, which are divided into five subscales: expressing happiness/excitement (HAP), expressing pride (GLO), managing anger/anger (ANG), managing depression/pain (DES), and managing guilt/shame (COM). Likert-5 points are used to score (ranging from 1= “very inconsistent” to 5 = “very consistent”). Higher scores indicate a higher level of the confidence in emotion regulation. In this study, the Cronbach’s α coefficient of the scale was 0.859. The Cronbach’s α coefficients of the five subscales were 0.605 (HAP), 0.448 (GLO), 0.779 (ANG), 0.759 (DES), and 0.617 (COM) respectively.

***Chinese Big Five Personality Inventory brief version, CBF-PI-B.***

The scale was developed by Wang Mengcheng et al. in 2011 [28]. It contains 40 items, which are divided into open subscale, rigor subscale, extroversion subscale, agreeableness subscale, and neuroticism subscale. This study uses the agreeableness subscale, a total of 8 items, using Likert-6 points to score (ranging from 1 = “completely inconsistent”, to 6 = “completely consistent”). Higher scores indicate a higher level of the pleasant traits of the participants. In this study, the Cronbach’s α coefficient of the scale was 0.769.

***Prosocial Tendencies Measure, PTM.***

Compiled by Carlo et al. in 2002 and revised by Kou et al. [29], it contains 26 items, which are divided into six subscales: open tendency (Pub), anonymity tendency (Ano), altruistic tendency (Alt), compliance tendency (Com), emotional tendency (Emo) and urgency tendency (Dir). Likert-5 points are used (ranging from 1 = “very unlike me” to 5 ="very much like me”). Higher scores indicate a higher level of the prosocial tendency. In this study, the Cronbach’s α coefficients of the six subscales were 0.802 (Pub), 0.840 (Ano), 0.791 (Alt), 0.773 (Com), 0.831 (Emo) and 0.677 (Dir), respectively.

## Results

### Confirmatory factor analysis

**Fig. 1:**
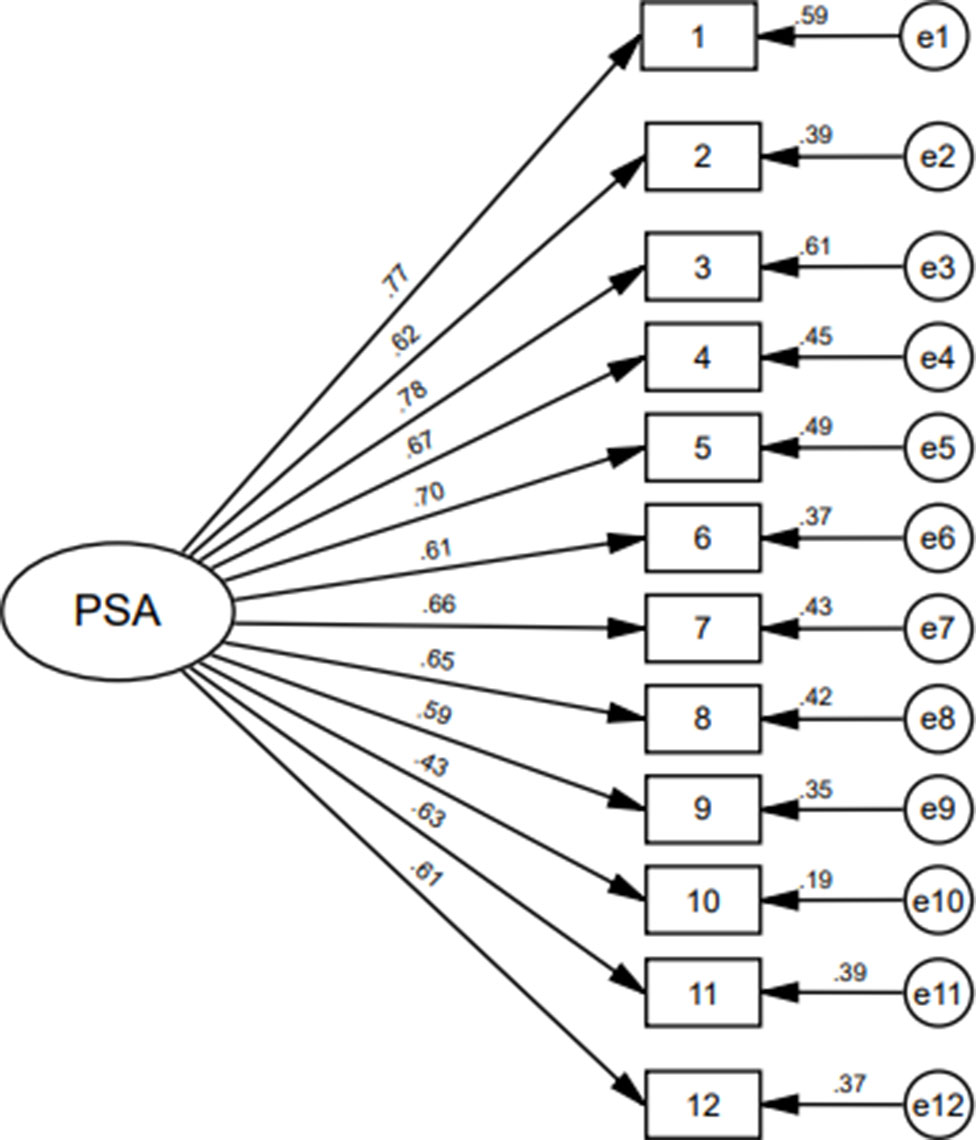
Load of scale items on single factor model

In order to test the factor structure of the scale, confirmatory factor analysis with the maximum likelihood estimation method was carried out by using AMOS. The results show that each fitting index accords with the statistical standard (see Table 4), indicating that the structure of the Chinese version of the 12-item PSA fits well and has good construct validity. The fitting index of the model is shown in Table 4 and the load of each item on the single-factor model is shown in Fig. 1.

**Table 4:** Analysis of validity factors of the revised model

model	χ2	df	χ2/df	RMSEA	SRMR	CFI	NFI	AGFI	TLI	IFI	GFI
12	225.711	54	4.180	0.074	0.042	0.937	0.919	0.907	0.922	0.937	0.936

### Concurrent validity

The Scale of School Adjustment for College Students (SSACS), the Scale of Regulatory Emotional Self-Efficacy (SRESE), the Chinese Big Five Personality Inventory brief version of the agreeableness subscale (CBF-PI-B), and the Prosocial Tendency Measure (PTM) were selected as the tools to measure the concurrent validity.

According to the literature, prosocial behavior has a significant positive correlation with school adaptation and agreeableness [30]. Emotion-regulated self-efficacy is not only positively correlated with prosocial behavior but also can predict the level of prosocial behavior [31]. The close relationship between prosocial tendency and prosocial behavior can be used as an indicator of prosocial behavior [32]. According to Table 5, the scores of prosocial items were significantly positively correlated with each criterion, and the correlation coefficient ranged from 0.394 to 0.619 (p < 0.01).

**Table 5:** Correlations of PAS, SSACS, CBF-PI-B, SRESE, PTM

	SSACS	CBF-PI-B	SRESE	PTM
PSA	0.429**	0.456**	0.394**	0.619**

Note: *P < 0.05, **P < 0.01, ***P < 0.001. PSA = Prosocialness Scale for Adults, SSACS = the Scale of School Adjustment for College Students; CBF-PI-B = the Chinese Big Five Personality Inventory brief version Subscale; SRESE = the Scale of Regulatory Emotional Self-Efficacy; PTM = the Prosocial Tendencies Measure

### Internal consistency analysis

The internal consistency reliability of Study 2 was tested. The results showed that the internal consistency coefficient of the Chinese version of the prosocialness scale for adults (PSA) was 0.890.

## Study 3

### Method

#### Participants and procedures

Four weeks after sending out the questionnaire in Study 2, 230 subjects were randomly selected to retest the 12-item version of the Prosocialness Scale for Adults (PSA). 223 valid questionnaires were collected, with an effective rate of 96.95%. Among them, there were 113 males (50.7%) and 110 females (49.3%). The age of the sample ranges from 17 to 23 years old, with an average age of 19.21 ± 1.21 years old. The academic year included 72 first-year college students (freshmen), 67 s-year college students (sophomores), 43 third-year college students (juniors), and 41 fourth-year college students (seniors).

The Chinese version of the Prosocialness Scale for Adults was issued. Before the test, a researcher read the instructions aloud, sent out the questionnaires, and collected the questionnaires uniformly after completion.

#### Measures

The 12-item version of the Chinese version of the Prosocialness Scale for Adults (PSA) was determined in Study 1 and 2.

#### Result

The test-retest data after 4 weeks showed that the test-retest correlation coefficient of the Chinese version of the Prosocialness Scale for Adults (PSA) was 0.801.

#### Discuss

The results of the item analysis of this study showed that the correlation between the items of the Prosocialness Scale for Adults (PSA) and the total score of the scale was between 0.587 and 0.732, and all reached the level of statistical significance, indicating that the discrimination of each item of the scale was good. The results of confirmatory factor analysis show that the scale has good structural validity and the single factor model fits well, which meets the requirements of psychometrics. It shows that the scale has good applicability in investigating the level of prosocial behavior.

Through item analysis, exploratory factor analysis and confirmatory factor analysis, the Chinese version of the Prosocialness Scale for Adults is formed, which includes 12 items. The scale is single-dimensional (the cumulative explanation rate is 48.305%). This is consistent with the theoretical basis of the original scale, indicating that there is cross-cultural consistency in the structure of prosocial behavior, and further verifies the stability of the structure of the adult prosocial scale. Item 12 (I tend to think of those who are uncomfortable) is deleted due to the low K value. In the factor analysis, items 8 (I can strongly feel the feelings of others), 14 (I am happy to share any good opportunities that come to me with friends), and 16 (even if my friend didn’t tell me directly. I can also immediately feel that he is uncomfortable) are deleted because of the high load on two factors.

In terms of concurrent validity, related studies have shown that prosocial behavior is related to a variety of good traits, such as agreeableness [33, 34], adaptability [8], and so on.

The results of the reliability analysis show that the adult prosocial scale has good reliability. The internal consistency coefficient of the total scale was 0.890. After 4 weeks, the test-retest reliability of the total scale was 0.801, and the cross-time stability was high, showing good measurement requirements.

In this study, the total score of the Prosocialness Scale for Adults (PSA) was significantly positively correlated with agreeableness, school adaptation, emotional expression self-efficacy, and prosocial tendency, which was consistent with the results of previous studies [35, 36], indicating that the revised Prosocialness Scale for Adults has good validity.

## Limitations and future research

Although there are some contributions to this study, there are also some limitations. The study on the validity and reliability of the Prosocialness Scale for Adults was conducted on a small sample of undergraduates. Therefore, more studies needed to be conducted on larger samples with participants from different backgrounds. In addition, the test-retest reliability of this study only tests the 4-week interval, therefore the study needs to test the reliability over a longer period. Further, in this study, the data were collected by using self-reporting instruments. In later studies, non-self-reported measurement instruments can be used to test the validity of the scale, to eliminate the influence of social approval tendency. Additionally, future studies can examine whether the Prosocialness Scale for Adults can predict other types of help behavior (volunteering, caretaking, peer supporting, etc.).

## Conclusion

Generally speaking, the development of a scale is a continuous process, and our research provides evidence for the reliability and validity of the Prosocialness Scale for Adults. The scale has good psychometric characteristics, including factor validity, simultaneous validity, internal reliability, and test-retest reliability. Therefore, the Prosocialness Scale for Adults is suitable for the evaluation of prosocial level of college students in China.

## Electronic supplementary material

Below is the link to the electronic supplementary material.

Supplementary Material 1

## Data Availability

The datasets generated and/or analyzed during the current study are not publicly available due to the subjects’ private information were collected, but are available from the corresponding authors on reasonable request.

## Abbreviations

AGFI: Adjusted Goodness of Fit Index
CBF-PI-B: Chinese Big Five Personality Inventory brief version subscale
CFI: Comparative fit index
GFI: goodness-of-fit index
IFI: incremental fit index
KMO: Kaiser–Meyer–Olkin
NFI: non-normed fit index
PSA: Prosocialness Scale for Adults
PTM: Prosocial Tendencies Measure
RMSEA: Root-mean-square errors of approximation
SRESE: the Scale of Regulatory Emotional Self-Efficacy
SRMR: Standardized root mean squared residual
SSACS: The Scale of School Adjustment for College Students
TLI: Tucker-Lewis index

## References

[1] Wispé LG (1972). Positive forms of Social Behavior: an overview. J Soc Issues.

[2] Yi L (2015). The cultivation of college students’ prosocial behavior from the perspective of moral education in Colleges and Universities. J Chongqing Univ Educ.

[3] Kou Y, Wang L. A Review of the Research on Children’s prosocial behavior and its intervention.Psychological Development and Education. 2003;86–91.

[4] Hofmann V, Müller CM (2018). Avoiding antisocial behavior among adolescents: the positive influence of classmates’ prosocial behavior. J Adolesc.

[5] Yates M, Youniss J (1996). A developmental perspective on Community Service in Adolescence. Soc Dev.

[6] Laible DJ, Carlo G, Roesch SC (2004). Pathways to self-esteem in late adolescence: the role of parent and peer attachment, empathy, and social behaviours. J Adolesc.

[7] Campbell L, Gulas CS, Gruca TS (1999). Corporate giving behavior and decision-maker social consciousness. J Bus Ethics.

[8] Zhu MX, Zhang WJ, Cai D (2016). The relationship between Social Support Network and Pro-social Behavior in Junior: the moderating effect of gender. China J Health Psychol.

[9] Coulombe BR, Yates TM (2018). Prosocial pathways to positive adaptation: the mediating role of teacher-child closeness. J Appl Dev Psychol.

[10] Flouri E, Sarmadi Z (2016). Prosocial behavior and childhood trajectories of internalizing and externalizing problems: the role of neighborhood and school contexts. Dev Psychol.

[11] Yu PL, Fan Q, Zhao MM, Liu HY, Chen B (2020). The relationship between parenting style and Prosocial Behavior of College students: the moderating effect of only-child or not. J Yanan Univ (Natural Sci Edition).

[12] Carlo G, Randall BA (2002). The development of a measure of Prosocial Behaviors for late adolescents. J Youth Adolesc.

[13] Qian XB, Gui SC. The Prosocial Behavior of College Students and Its Training.Journal of Jiangsu Radio & Television University. 2006;79–81.

[14] Hong HF, Kou Y. The Further Study on the Correlations of Prosocial Tendencies and Prosocial Moral Reasoning with Canonical Correlation Analysis.Psychological Development and Education. 2008;113–8.

[15] Yu K, Ma Y, Tan C. Chinese College Students’ Prosocial Tendencies, Prosocial Moral Reasoning and Their Correlation Patterns.Psychological Science. 2004;329–32.

[16] Caprara GV, Capanna C, Steca P, Paciello M (2005). Misura e determinanti personali della prosocialità. Un approccio sociale cognitivo. GP.

[17] Caprara GV, Steca P, Zelli A, Capanna C (2005). A New Scale for measuring adults’ prosocialness. Eur J Psychol Assess.

[18] Anlı G (2019). Adaptation of the prosocial behavioral intentions scale for use with turkish participants: assessments of validity and reliability. Curr Psychol.

[19] Martínez-Pampliega A, Cormenzana S, Sanz M, Barni D, Simon J, Alomar E (2018). Metric Goodness of the adult prosocialness scale. Comparative study of Italy and Spain. Span J Psychol.

[20] Martínez RS. Relación entre conductas prosociales y participación en grupos online en jóvenes con discapacidad motora. Relationship between prosocial behaviour and participation in online support groups for young people with motor disability. 2017; 17:57–66.

[21] Yuan ML, Li WQ, Kou Y. Social Class and Prosocial Behavior: how and why social class affects prosocial behavior. JOURNAL OF BEIJING NORMAL UNIVERSITY(SOCIAL SCIENCES); 2019. pp. 37–46.

[22] Abdullah AA, Hamsan HH, Ma’rof AA (2020). How do personality factors associate with Prosocial Behavior? The Mediating Role of Empathy. IJARBSS.

[23] Penner LA (2005). Dovidio. JF, Piliavin. JA, Schroeder. DA. Prosocial Behavior: Multilevel Perspectives. Ann Rev Psychol.

[24] Guo Y (2017). The influence of Social Support on the Prosocial Behavior of College students: the mediating Effect based on Interpersonal Trust. Engl Lang Teach.

[25] Bierhoff HW, Klein R, Kramp P (1991). Evidence for the altruistic personality from data on Accident Research. J Pers.

[26] Hou J (2014). Development of the scale of School Adjustment for College Students. China J Health Psychol.

[27] Wang YJ, Dou K, Liu Y. Revision of the Scale of Regulatory Emotional Self-efficacy. Journal of Guangzhou University (Social Science Edition). 2013; 12:45?50.

[28] Wang MC, Dai XY, Yao SQ. Development of the Chinese Big Five Personality Inventory (CBF-PI) III: Psychometric Properties of CBF-PI Brief Version. Chinese Journal of Clinical Psychology. 2011; 19:454–7.

[29] Kou Y, Hong HF, Tan C, Li L. Revisioning Prosocial Tendencies Measure for Adolescent.Psychological Development and Education. 2007;112–7.

[30] Chen FX, Fan FM (2014). Relationships among College Adjustment, Resilience and Mental Health in Freshmen. China J Health Psychol.

[31] Caprara GV, Scabini E, Barbaranelli C, Pastorelli C, Regalia C, Bandura A (1999). Perceived emotional and interpersonal self-efficacy and good social functioning. Giornale Italiano di Psicologia.

[32] Zhan CM, Zou H, Hou K (2006). The relationship between Primary School Children’s respect behavior, personality and class environment. Psychol Dev Educ.

[33] He MY. A Summary of the present situation of the Research on Children’s prosocial Development.Modern educational science. 2015;23–6.

[34] Pursell GR, Laursen B, Rubin KH, Booth-LaForce C, Rose-Krasnor L (2008). Gender differences in patterns of association between prosocial behavior, personality, and externalizing problems. J Res Pers.

[35] Caprara GV, Steca P (2006). The Contribution of Self–Regulatory Efficacy Beliefs in managing Affect and Family Relationships to positive thinking and hedonic balance. J Soc Clin Psychol.

[36] van Emmerik IJH, Jawahar IM, Stone TH (2004). The relationship between personality and discretionary helping behaviors. Psychol Rep.

