# Supplementary material for: Revision and validation of the prosocialness scale for adults (PSA) among chinese college students
Source: BMC Psychol. 2023 Apr 19;11:124. doi: 10.1186/s40359-023-01124-3 (PMC10114401; doi:10.1186/s40359-023-01124-3)
Supplement: Supplementary file 1 — Supplementary Material 1 [file 40359_2023_1124_MOESM1_ESM.pdf]

Final Items of the Chinese version of the Prosocialness Scale for Adults (PSA)

1.I am happy to help my friends or colleagues in the activities.

2.I would like to share my things with my friends.

3.I try to help others.

4.I can help those in need in voluntary activities.

5.I am very warm-hearted to those who need help.

6.I will help those in need immediately.

7.I will try my best to help others avoid getting into trouble.

8.I would like to benefit others with my knowledge and ability.

9.I try to comfort those who are sad.

10.It’s easy for me to lend money or other things.

11.I try to get close to and take care of those in need.

12.I will spend time with friends who feel lonely.
